# Oridonin-induced ferroptosis and apoptosis: a dual approach to suppress the growth of osteosarcoma cells

**DOI:** 10.1186/s12885-024-11951-1

**Published:** 2024-02-12

**Authors:** Feifan Zhang, Yang Hao, Ning Yang, Man Liu, Yage Luo, Ying Zhang, Jian Zhou, Hongjian Liu, Jitian Li

**Affiliations:** 1 Hunan University of Chinese Medicine, Changsha, China; 2 Henan Luoyang Orthopedic Hospital (Henan Provincial Orthopedic Hospital), Zhengzhou, China; 3 Henan University of Chinese Medicine, Zhengzhou, China; 4 Qingdao Hospital, University of Health and Rehabilitation Sciences (Qingdao Municipal Hospital), Qingdao Municipal Hospital, Qingdao, China; 5 Department of Orthopedics, The First Affiliated Hospital of Zhengzhou University, Zhengzhou, China

**Keywords:** Osteosarcoma, Oridonin, Apoptosis, Ferroptosis, Anti-tumor, ROS

## Abstract

**Background:**

Osteosarcoma (OS) is one of the most common aggressive bone malignancy tumors in adolescents. With the application of new chemotherapy regimens, finding new and effective anti-OS drugs to coordinate program implementation is urgent for the patients of OS. Oridonin had been proved to mediate anti-tumor effect on OS cells, but its mechanism has not been fully elucidated.

**Methods:**

The effects of oridonin on the viability, clonal formation and migration of 143B and U2OS cells were detected by CCK-8, colony formation assays and wound-healing test. Kyoto Encyclopedia of Genes and Genomes (KEGG) enrichment analysis was used to explore the mechanism of oridonin on OS. Western blot (WB), real-time quantitative PCR (qRT-PCR) were used to detect the expression levels of apoptosis and ferroptosis-relative proteins and genes. Annexin V-FITC apoptosis detection kit and flow cytometry examination were used to detect the level of apoptosis. Iron assay kit was used to evaluate the relative Fe^2+^ content. The levels of mitochondrial membrane potential and lipid peroxidation production was determined by mitochondrial membrane potential detection kit and ROS assay kit.

**Results:**

Oridonin could effectively inhibit the survival, clonal formation and metastasis of OS cells. The KEGG results indicated that oridonin is associated with the malignant phenotypic signaling pathways of proliferation, migration, and drug resistance in OS. Oridonin was capable of inhibiting expressions of BAX, cl-caspase3, SLC7A11, GPX4 and FTH1 proteins and mRNA, while promoting the expressions of Bcl-2 and ACSL4 in 143B and U2OS cells. Additionally, we found that oridonin could promote the accumulation of reactive oxygen species (ROS) and Fe^2+^ in OS cells, as well as reduce mitochondrial membrane potential, and these effects could be significantly reversed by the ferroptosis inhibitor ferrostatin-1 (Fer-1).

**Conclusion:**

Oridonin can trigger apoptosis and ferroptosis collaboratively in OS cells, making it a promising and effective agent for OS therapy.

**Supplementary Information:**

The online version contains supplementary material available at 10.1186/s12885-024-11951-1.

## Introduction

Osteosarcoma (OS), a common bone malignancy in adolescents, has a high mortality and disability rates [1]. Surgical resection was a primary method for treatment of OS in the past, and the use of neoadjuvant chemotherapy has raised the survival rate to 60–70% [2]. However, in recent years, chemotherapy causes great physical and psychological suffering to patients [3]. Meanwhile, the emergence of multi-drug resistance poses a serious challenge to the chemotherapy regimen [4, 5], targeting drug resistance-related mechanisms may be one of the keys to OS therapy [6]. Therefore, solving the problem of chemotherapeutic drug resistance is crucial for the treatment of OS.

With the advantages of multi-target and multi-pathway treatment increasing [7], the traditional Chinese medicine can slow the growth of cancers, reduce drug resistance and lessen the negative effects from radiotherapy and chemotherapy significantly [8], which had become one of the key directions of anti-tumor drug research. *Rabdosia rubescens*, a traditional Chinese herbal medicine with the functions of clearing heat, detoxifying, activating blood circulation and anti-cancer, which had been used in the treatment of chronic bronchitis, chronic hepatitis, esophageal cancer, liver cancer, breast cancer, and other diseases [9–11]. Oridonin, the primary anticancer component in *Rabdosia rubescens*, was demonstrated to inhibit the proliferation and metastasis of OS while increasing the sensitivity of OS to doxorubicin, making it a promising drug for clinical treatment of OS [12–15]. However, the specific mechanism of inhibiting OS resistance is still unclear.

Studies have shown that programmed cell death (PCD) is closely related to chemotherapy resistance of cancers [16]. Apoptosis is the main kind of PCD, and tumor development, metastasis and drug resistance of OS cells were all closely related to apoptosis as reported [17, 18]. Inhibition of apoptosis, one of the classic markers of cancer, is related to drug resistance and may be an important therapeutic target to overcome the drug resistance [19]. Ferroptosis, a new form of PCD [20], characterized by hazardous lipid peroxidation and mitochondrial dysfunction [21], had been proved closely related to inhibition of cancer cells proliferation, migration, and invasion [22] Studies have shown that ferroptosis can affect the efficacy of cancer treatment and reverse the resistance to chemotherapy, targeted therapy, and immunotherapy by regulating GPX4 signaling pathways, iron metabolism, and lipid metabolism [16, 23, 24]. Activating ferroptosis to fight against cancer was a treatment with high safety and selectivity [25]. Meanwhile, there was correlation between apoptosis and ferroptosis [26, 27]. Multiple studies had shown that simultaneous targeting of apoptosis and ferroptosis can effectively bypassing chemoresistance, which was an effective strategy to inhibit OS cells [28–31]. Therefore, we hypothesize that oridonin can cause apoptosis and ferroptosis simultaneously, thereby enhancing its inhibitory effects on osteosarcoma proliferation, metastasis, and drug resistance.

In this study, we would explore the inhibitory effect of oridonin on the proliferation and migration of OS cells, as well as its promoting effect on apoptosis and iron death of OS cells, so as to provide a new idea for the treatment of OS.

## Materials and methods

### Cell culture

Human OS cell lines including 143B, U2OS were preserved by molecular biology laboratory, Henan Luoyang Orthopedic Hospital (Henan Provincial Orthopedic Hospital), (Henan, China). 143B and U2OS cells were cultured in McCoy’s 5A medium (#C3020–0500, VivaCell) which supplemented with 10% fetal bovine serum (#10270–106, Gibco) and 1% penicillin/streptomycin (#C3420–0100, VivaCell), and cultured at 37 °C in a humidified atmosphere with 5% CO2.

### Reagents and antibodies

Oridonin (HPLC ≥98%) was purchased from Shanghai yuanye Bio-Technology Co., Ltd. (#B20310), then the drug was dissolved in DMSO and stored in − 20 °C for usage. Ferrostatin-1 (Fer-1) was purchased from MedChemExpress (#HY-100579, MCE), and stored in − 20 °C for usage. All primary antibody used in the experiments included Actin (1:10000, #60008–1-Ig, Proteintech), Bcl-2 (1:1000, #61193, Proteintech), BAX (1:5000, #50599–2-Ig, Proteintech), cl-caspase3 (1:10000, #19677–1-AP, Proteintech), Tublin (1:1000, #66031–1-Ig, Proteintech), GAPDH (1:2000, #CL594–60004, Proteintech), GPX4 (1:5000, #67763–1-IG, Proteintech), SLC7A11 (1:1000, #26864–1-AP, Proteintech), FTH1 (1:5000, #11682–1-AP, Proteintech), and ACSL4 (1:10000, #81196–1-RR, Proteintech), they were stored in − 20 °C for usage. Ferrostatin-1 (Fer-1) (#HY-100579, MCE), an effective ferroptosis inhibitor [32], was dissolved in dimethylsulfoxide (DMSO) to create stock solutions and stored at − 20 °C.

### Cell viability assay

143B and U2OS cells were exposed to oridonin (0, 5, 10, 20 μM) for 24 h and 48 h in 96-well plates with a density of 7000 cells per well. Cell proliferation was detected by CCK-8 (#C0038, Beyotime) assays according to the manufactures instructions. The optical density (OD) value was detected by a microplate reader (#Epoch, BioTek). The IC50 value was calculated by non-linear regression fitted via GraphPad Prism (Version 9) software.

### Plate colony formation assays

143B and U2OS cells were exposed to oridonin (0, 5, 10 μM) for 10 days in 6-well plates with a density of 400 cells per well. When 143B and U2OS cells formed sufficiently large colonies, they were fixed with 4% paraformaldehyde (#BL539A, Biosharp) for 20 min at RT and stained with 0.5% crystal violet solution (#C8470, Solarbio) incubate for 2 min at RT. The colony numbers were counted under the microscope (#IX73, OLYMPUS).

### Wound-healing test

143B and U2OS cells were exposed to oridonin (0, 5, 10 μM) for 10 days in 6-well plates at a density of 5 × 10^4^. Each well was scraped a scratch with a 20 μL pipette tip vertically when the cells reached 100% confluence, then the plates were washed with PBS three times and changed to fresh serum-free medium. Microscope (#IX73, OLYMPUS) was used to image the cells in the same vision at hours 0, 12 and 24 h to observe and photograph the changes of scratch width. The migration rate was calculated by a formula: (scratch distance (0 h) – scratch distance (12 h / 24 h)) scratch distance (0 h).

### Kyoto encyclopedia of genes and genomes (KEGG) enrichment analysis

Potential targets of oridonin through DRUGBANK (https://go.drugbank.com/) database were searched, and 487 targets were obtained after deleting duplicates. 1263 correlated targets of OS were collected and filtered from Genecards (https://www.genecards.org/) database, OMIM (https://www.omim.org/) database, TTD (https://db.idrblab.net/ttd/) database, DisGENET (https://www.disgenet.org/) database. Intersection 115 targets of the two were imported into the DAVID (https://david.ncifcrf.gov/) database for KEGG enrichment analysis, resulting in 162 enrichment findings. Among these, 20 relevant signaling pathways were selected and displayed through the Microbiology Information Online platform (http://www.bioinformatics.com.cn/).

### Flow cytometry examination

143B and U2OS cells were cultured in 6-well plates for 24 h, after which they were divided into control and oridonin groups (10 μM and 20 μM), and then continued to be cultured for another 24 h before collecting the cells. The positive control group cells were incubated on ice with an Apoptosis Positive Control Solution (500 μL) for 30 minutes, followed by one wash with PBS. All groups were resuspended in 1 × Binding Buffer. The negative control group was divided into four tubes, three of which were respectively added with Annexin V-FITC (5 μL), propidium iodide (PI) (10 μL), or both, and incubated at RT in the dark for 5 minutes. The positive control and oridonin groups were simultaneously added with Annexin V-FITC (5 μL) and propidium iodide (PI) (10 μL), then incubated at room temperature in the dark for 5 minutes. Subsequently, cells from each group were subjected to apoptosis analysis using a FACScan flow cytometer (#FACSAria II, BD).

### Western blot analysis (WB)

143B and U2OS cells were exposed to oridonin (0, 5, 10 μM) for 24 h in 6-well plates cells. The cultures were discarded medium and washed with 1 × PBS (#G4202, Servicebio) three times. The 143B and U2OS cells were collected and mixed with cell lysates 80 uL (#P0013B, Beyotime) incubated for 1 h on ice, then sonicated and centrifuged (30 min, 14,000 g, 4 °C). BCA method was performed for the whole protein quantification of the cells by BCA kit (#PC0020, Solarbio). Via sodium dodecyl sulfate polyacrylamide gel electrophoresis and transmembrane, the 143B and U2OS cells’ protein was transferred onto polyvinylidene difluoride membranes. The membranes were incubated with the indicated antibodies prior to detection using enhanced chemiluminescent reagent. Then the membranes were blocked with 5% non-fat milk for 1 h at RT and incubated with the primary antibody overnight at 4 °C. After incubating the secondary antibody (anti-rabbit/mouse IgG HRP) for 1 h and wash it 10 min for three times at RT, the membranes were visualized by WB exposure machine (Amersham Imager 680/Tanon 4800 Multi) after treatment of ECL chemiluminescence kit (#P0018FS, Beyotime).

### Real-time quantitative PCR (qRT-PCR)

Total RNA was extracted by RNAzol reagent (#15596018, ThermoFisher) and reversed to cDNA (Takara). The qRT-PCR examination was performed by the Fluorescent quantitative PCR instrument (#CFX96 deep well, Biorad) using a SYBR Select Master Mix kit (#4472908, Life Technology) according to the instructions. The relative expressions of the target genes were normalized against GAPDH and analyzed using the 2^−△△Ct^ method [33]. Supplementary Table 1 displays the detailed sequences of primers.

### Assessment of Fe^2+^ content

143B and U2OS cells were exposed to oridonin (0, 5, 10 μM) for 24 h in 15 dish plates, after which the Iron assay Kit (#BC5415, Solarbio) was used to evaluate the relative Fe^2+^ content directed by the manufacturer. The value of OD in 593 nm was measured on microplate reader (#Epoch, BioTek). The standard curve was drawn against the standard samples, then the relative Fe^2+^ level was calculated.

### Measurement of ROS

After 143B and U2OS cells adhered, exposed them to the medium containing oridonin (0, 5 μM and 10 μM) Serum-free McCoy’s 5A for 24 h in 6-well plates Cells. ROS assay kit (#R6033, UElandy) was used to test the level of ROS which directed by the manufacturer. Before treating all cells with 1 mL the ROS fluorescent probe-2, 7-dichlorofuorescin diacetate (DCFH-DA, 10 μM) for 30 min at 37 °C avoid light, the positive control group was first treated with 1 mL ROS positive inducer-ROSUP (100 μM). Then 143B and U2OS cells were washed twice with serum-free cell culture medium, then viewed them under fluorescence microscope (#BX53, OLYMPUS).

### Measurement of mitochondrial membrane potential

Mitochondrial membrane potential detection kit (JC-1) (#J6004S, UElandy) was used to determine the mitochondrial membrane potential. Hoechest33342 (#H4047, UElandy) was used to tag the DNA of living cells. When 143B and U2OS cells adhered, exposed them to oridonin (0, 5 μM and 10 μM) for 24 h in 6-well plate. After removing the media, 143B and U2OS cells were washed with PBS once. 500 μL serum-free cell culture medium, 500 μL JC-1 solution and 500 μL Hoechest33342 were added to each well, the positive control group was additionally treated with 500 μL CCCP (50 μM), then mixing them and incubated for 20 min. The 143B and U2OS cells were washed twice by JC-1 dye buffer after incubation, then added 2 ml cell culture medium to it. Cells were observed under fluorescence microscope (#BX53, OLYMPUS).

### Statistical analysis

Image J software was used to analysis the results of WB. GraphPad Prism (Version 9) was operated to evaluate all data, which were shown as the mean ± SD. Two-sample *t* test was used for independent samples and one-way analysis of variance (ANOVA) was used for multiple samples. The differences were considered statistically significant with a significant level (*p*) less than 0.05.

## Results

### Oridonin inhibited the proliferation and migration of OS cells

**Fig. 1:**
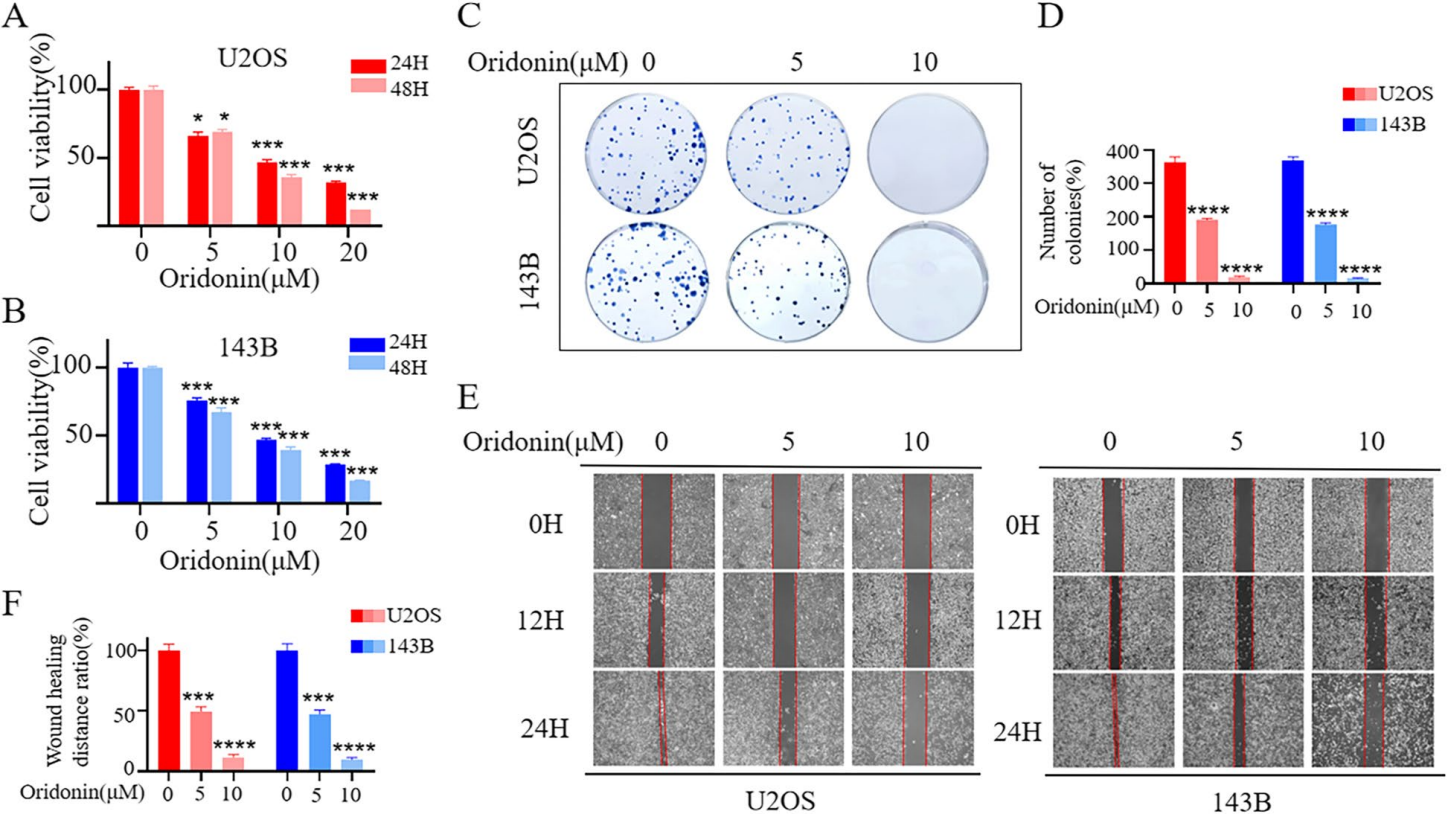
Oridonin inhibits OS cells viability. **A**-**B** 143B and U2OS cells lines were treated with oridonin (0, 5, 10 and 20 μM) for 24 and 48 h. Cell viability was measured by CCK-8 assays. (*n* = 5, * *p* < 0.05, ** *p* < 0.01, *** *p* < 0.001, **** *p* < 0.0001, vs. normal). **C**-**D** 143B and U2OS cells treated with oridonin (0, 5, 10 μM). Colony formation was evaluated by colony formation assays. (*n* = 3, **** *p* < 0.0001, vs. normal). **E**-**F** 143B and U2OS cells lines were treated with oridonin. (0, 5, 10 μM) for 0 h, 12 h and 48 h. Cells migration was evaluated by wound-healing tests (4X). (n = 3, *** *p* < 0.001, **** *p* < 0.0001, vs. normal)

To discover the anti-cancer effects of oridonin on cell viability of OS cells, 143B and U2OS cells were treated at indicated time points (24 or 48 h) with various concentration of oridonin (0, 5 μM, 10 μM and 20 μM). Our results indicated that oridonin exhibited significant cytotoxic effects to OS cells, the cell viability of 143B and U2OS cells was inhibited in a dose-dependent manner compared to the untreated group (Fig. 1A, B). The half maximal inhibitory concentrations (IC50) of oridonin on these cells at 24 h were 9.716 μM and 10.68 μM respectively. We used 143B and U2OS cells with of oridonin (5 and 10 μM) treatment in the following experiments. The result of plate colony formation assays (Fig. 1C, D) and wound healing test (Fig. 1E, F) demonstrated that the ability of OS cells proliferation and migration were controlled by oridonin. In conclusion, these results suggested that oridonin may dose-dependently attenuated the proliferation and metastasis of OS cells.

### Oridonin induced apoptosis in OS cells

**Fig. 2:**
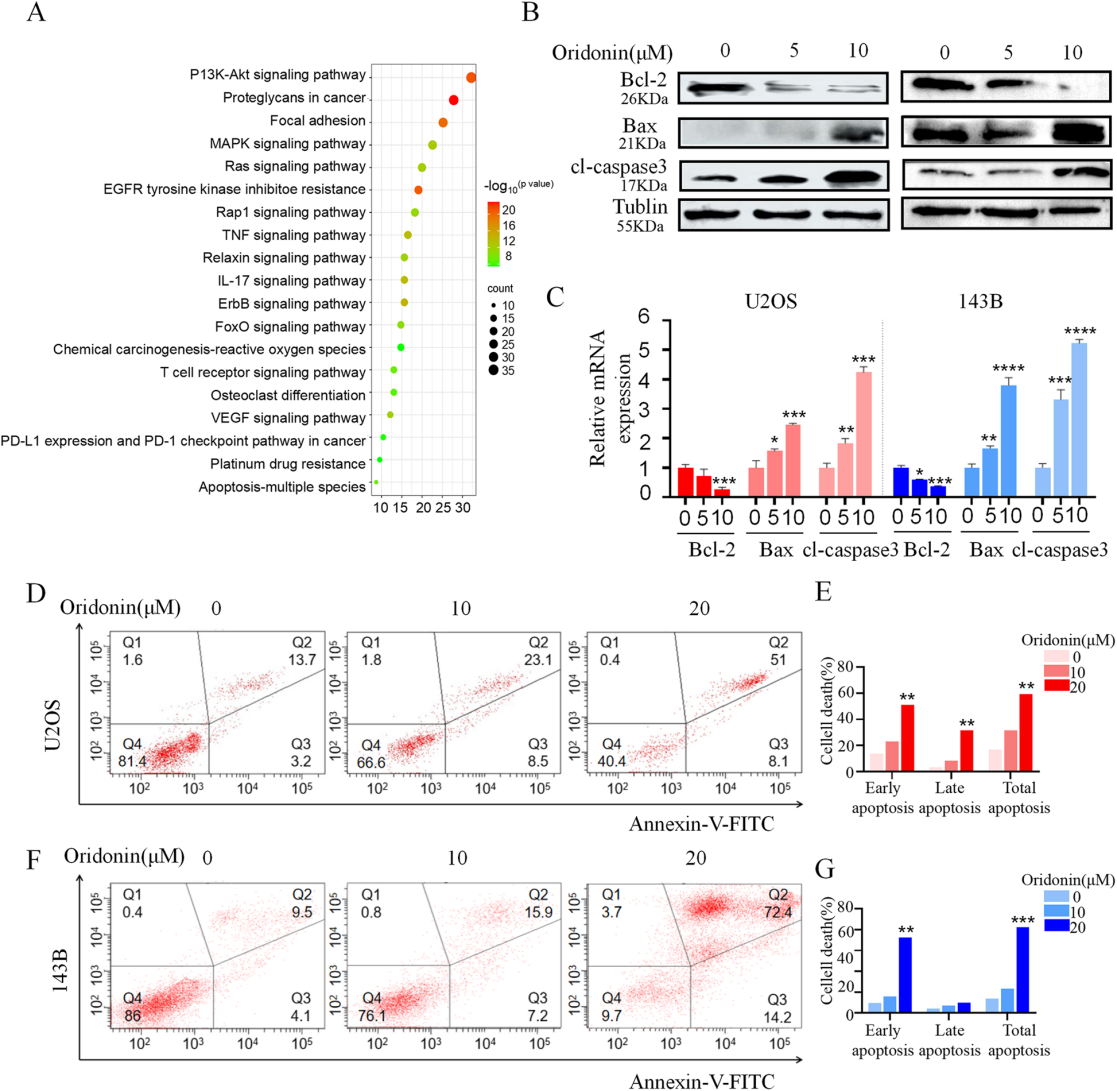
Oridonin induced apoptosis in OS cells. **A** The enrichment analysis of KEGG pathways resulted in 162 pathways (*p* value < 0.05), select 20 of them to draw the signal path bar chart. **B-C** WB and qRT-PCR was used to analysis the expression levels of Bcl-2, Bax and cleaved caspase3 in 143B and U2OS cells exposed to oridonin (0–5-10 μM) for 24 h, Tublin and GAPDH as reference (n = 3, * *p* < 0.05, ** *p* < 0.01, *** *p* < 0.001, **** *p* < 0.0001, vs. normal). **D-G** The 143B and U2OS cells were treated with oridonin (0–10-20 μM) for 24 h, detected apoptotic cells by Annexin V-FITC and PI double staining and quantified (n = 3, * *p* < 0.05, ** *p* < 0.01, *** *p* < 0.001, **** *p* < 0.0001, vs. normal)

Based on the results of the KEGG enrichment analysis (Fig. 2A), we discovered that oridonin not only affects the signal pathways related to proliferation and metastasis of OS cells, but also participates in the tumor immunity and drug resistance of OS cells, such as EGFR tyrosine kinase inhibitor resistance, platinum drug resistance, T cell receptor signaling pathway and anti-programmed cell death 1 (PD-L1) expression and programmed death-ligand 1 (PD-1) checkpoint pathway in cancer. To further explore the possible mechanism of oridonin’s involvement in tumor suppression and drug resistance, we first examined the effect of oridonin on apoptosis of 143B and U2OS cells. 143B and U2OS cells were subjected to oridonin (0, 5 μM and 10 μM) for 24 h before monitoring the markers associated with apoptosis. The results of WB and qRT-PCR demonstrated that pro-apoptotic Bax and cleaved caspase-3 were increased, while anti-apoptotic Bcl-2 expression was clearly inhibited (Fig. 2B, C). The results of apoptosis investigations using flow cytometry revealed that when 143B and U2OS cells were exposed to oridonin (0, 10 μM and 20 μM) for 24 h, the percentage of apoptotic cells rose in a concentration-dependent way (Fig. 2D-G). In general, our data demonstrated that oridonin increased apoptosis in OS cells.

### Oridonin triggered the ferroptosis in OS cells

**Fig. 3:**
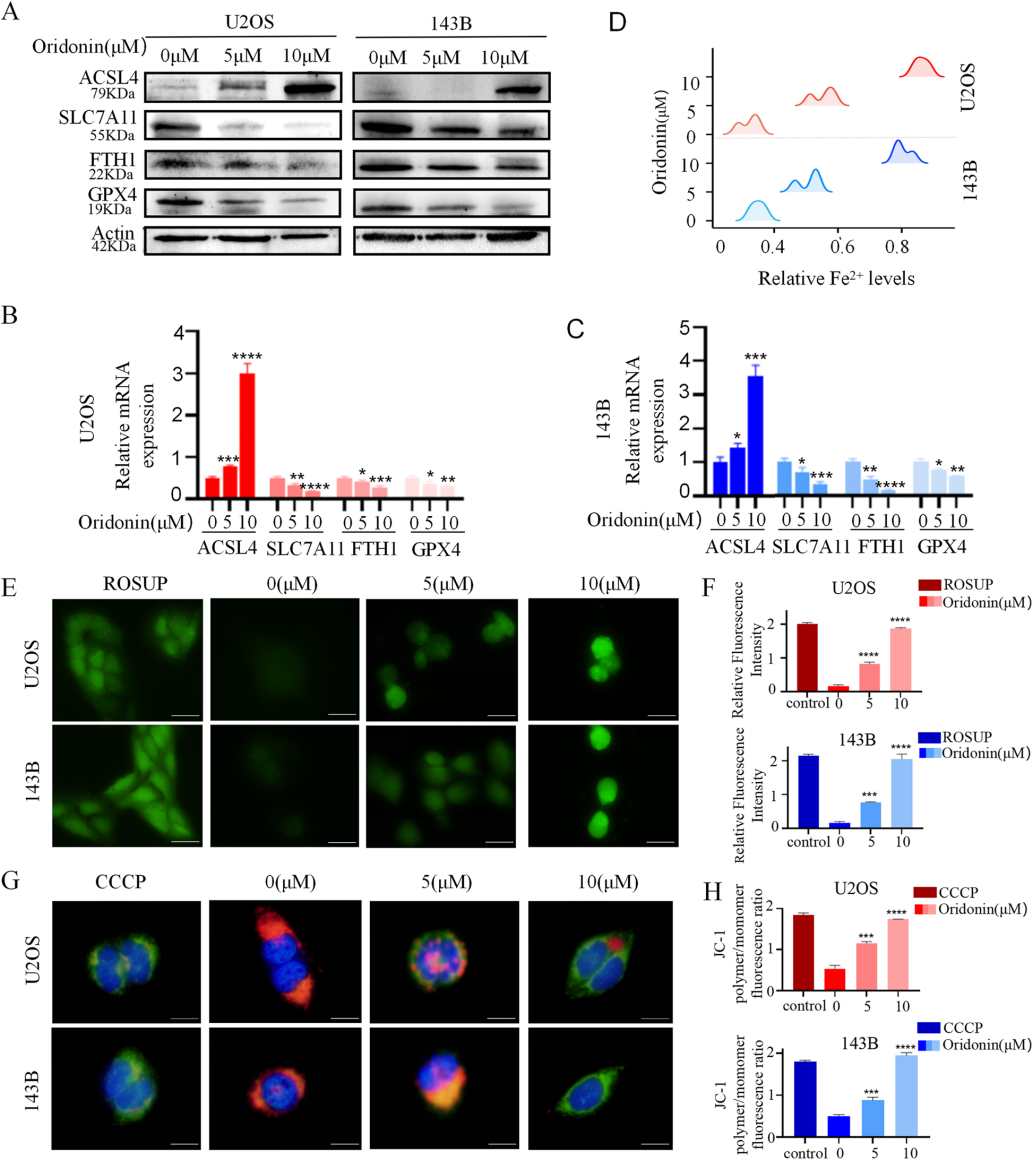
Oridonin could triggered the ferroptosis in OS cells. **A-C** The expressions of ACSL4, SLC7A11, FTH1 and GPX4 in oridonin treated U2OS and 143B cells analyzed by WB and qRT-PCR, Actin and GAPDH as reference. (n = 3, * *p* < 0.05, ** *p* < 0.01, *** *p* < 0.001, **** *p* < 0.0001, vs. normal). **D** The accumulation of Fe^2+^ was detected in the oridonin treated cells by a microplate reader. (*p < 0.05, ** *p* < 0.01, *** *p* < 0.001, **** *p* < 0.0001, vs. normal). **E-F** ROS were detected by DCFH-DA probe in the oridonin treated cells, the accumulation of ROS was illustrated by green fluorescence (40X). Cells treated with ROSUP were used as positive control (*p < 0.05, ** *p* < 0.01, *** *p* < 0.001, **** *p* < 0.0001, vs. normal). **G-H** The mitochondrial membrane potential detected by JC-1 probe, dye Hoechest33342 was used for nuclear localization (100X). Cells treated with CCCP were used as positive control (*p < 0.05, ** *p* < 0.01, *** *p* < 0.001, **** *p* < 0.0001, vs. normal)

Studies have shown that ferroptosis is related to tumor inhibition, T cell immunity and PD-L1/ PD-1 treatment resistance, etc. [34, 35]. Therefore, the study further explored the effect of oridonin on ferroptosis of OS cells. The SLC7A11, GPX4, FTH1 and ACSL4 were all strongly associated with the occurrence of ferroptosis, and we therefore examined their expression in OS cells treated with oridonin (0, 5 μM and 10 μM) for 24 h. The results of WB and qRT-PCR revealed that oridonin could dramatically downregulate the expression of SLC7A11, GPX4, and FTH1 expression, while upregulate the expression of ACSL4 (Fig. 3A-C). Ferrous ion colorimetry was used to determine the intracellular iron. Compared with the untreated group, Fe^2+^ level was increased in oridonin-treated groups (5 μM and 10 μM) with dose-dependent performance (Fig. 3D). In addition, we observed that the green fluorescence obviously increased in a dose-dependent approach, illustrating that oridonin increased the ROS accumulation (Fig. 3E, F) and reduced the mitochondrial membrane potential (Fig. 3G, H) in these two OS cells. These results supported the fact that oridonin might induced the ferroptosis in OS cells.

### Oridonin promoted OS cells death may in a ferroptosis-dependent manner

**Fig. 4:**
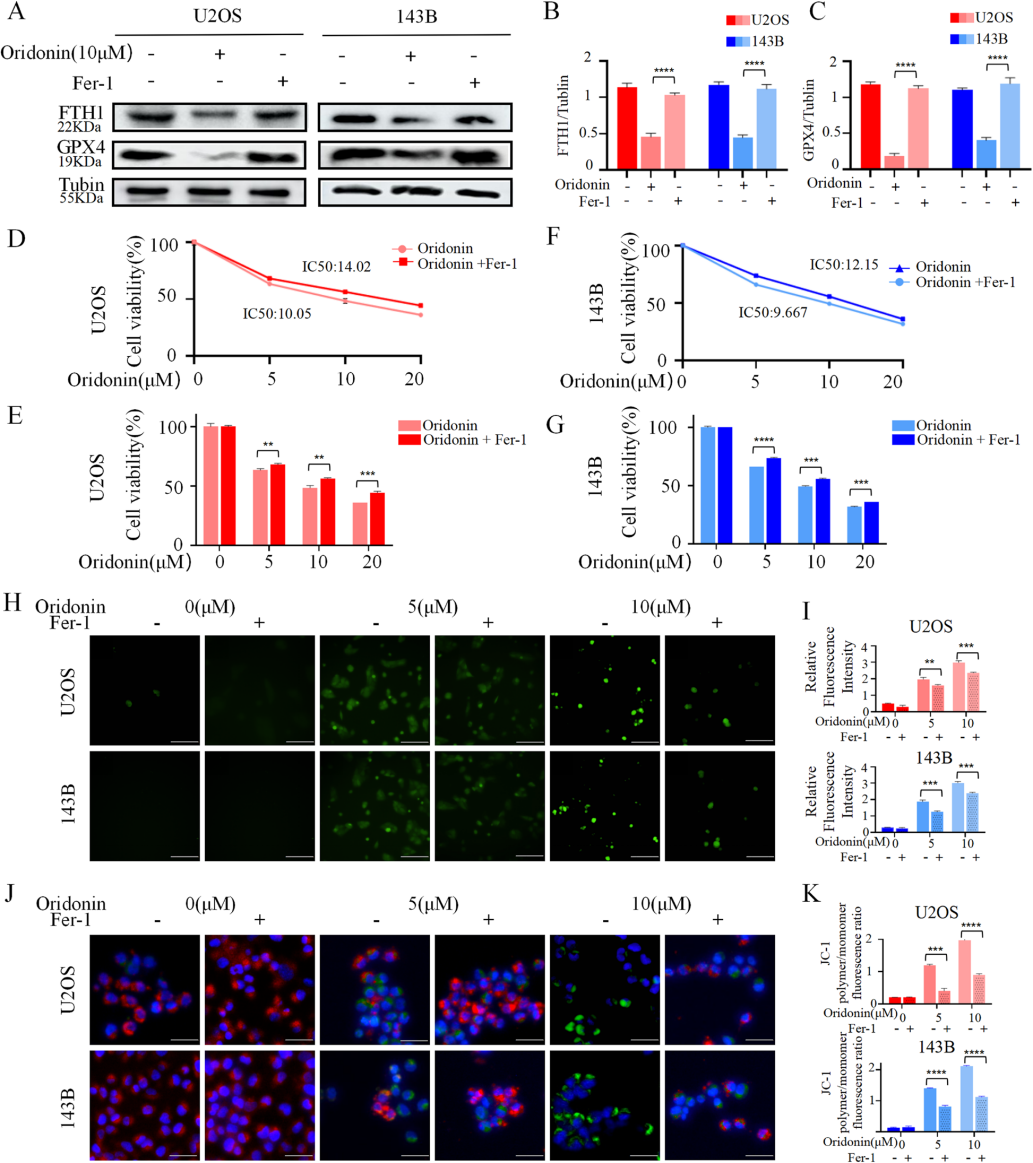
Fer-1 rescued the anti-tumor effects of oridonin in OS cells. **A**-**C** The expressions of ferroptosis markers FTH1 and GPX4 in oridonin-treated 143B and U2OS cells were increased after added Fer-1. Actin as reference. (n = 3, * p < 0.05, ** *p* < 0.01, *** *p* < 0.001, ****p < 0.0001, vs. normal). **D**-**G** The 143B and U2OS cells were co-treated with oridonin and Fer-1, and the cell viability was examined by CCK-8 assays. (n = 3, * *p* < 0.05, ** *p* < 0.01, *** *p* < 0.001, **** *p* < 0.0001, vs. normal). **H-K** Compared the intracellular contents of ROS (20X) (**H-I**) in the oridonin and co-treated OS cells, meanwhile, observed the change of mitochondrial membrane potential (40X) (**J-K**). Cells treated with ROSUP and CCCP were used as positive control (n = 3, * *p* < 0.05, ** *p* < 0.01, *** *p* < 0.001, **** *p* < 0.0001, vs. normal)

To demonstrate the role of ferroptosis in the anti-OS activity of oridonin, 143B and U2OS cells were pretreated with Fer-1 (1 μM) to suppress the ferroptosis generated by oridonin in OS cells. The upregulation of FTH1 and GPX4 relevant to oridonin were rescued by Fer-1 (Fig. 4A-C). Fer-1 could relieve the oridonin-mediated cytotoxicity, increased the IC50 value of oridonin act on 143B and U2OS cells to 12.15 μM and 14.02 μM, respectively (Fig. 4D-G). Meanwhile, Fer-1 inhibited oridonin-induced intracellular ROS accumulation (Fig. 4H, I) and reversed the mitochondrial membrane potential suppression (Fig. 4J, K). Collectively, the results presented above suggested that oridonin-triggered ferroptosis might be one of the primary mechanisms by which oridonin suppressesd the growth of OS cells.

## Discussion

OS is one of the most common aggressive bone malignancy tumors in the adolescence, with a significant mortality and disability rate. Despite advances in conventional and targeted therapies, the survival rate of OS has not improved significantly in last ten years [36]. Each year, approximately 2–3 million people died from OS on a global scale [2], the high metastatic potential and therapy resistance of OS cited as the primary causes [37]. Therefore, in this study, we focused on finding a drug which can simultaneously inhibit the proliferation, metastasis and drug resistance of OS.

Previous studies have demonstrated that the traditional Chinese medicine treatment has the advantage of being multi-target and multi-pathway, which has distinct advantages in drug resistance and tumor metastasis inhibition [38]. Oridonin is derived from the traditional Chinese herb *Rabdosia rubescens* and had been shown to have a wide variety of anticancer effects and was regarded a promising antitumor agent [39–41]. Network pharmacology had a certain usefulness in tumor therapy mining [42, 43]. In the study, network pharmacology was applied to explore the potential pathways of oridonin and the results have showed that oridonin was associated with multiple phenotypes and pathways associated with proliferation and metastasis of OS. Then we further searched the relevant literature and found that the previous studies have fully investigated the mechanism of oridonin in inhibiting the proliferation and metastasis of OS. Yang et al. found that oridonin inhibited 143B cells proliferation via upregulating the Dkk-1 expression and/or enhancing the function of GSK3β to downregulate Wnt/β-catenin signal transduction [15]. Yang et al. found that oridonin inhibited TGF-β-induced phosphorylation of Smad 2/3 and prevented Smad dimer translocation into the nucleus to inhibit the metastasis of OS cells [44]. In addition, Song et al. and Xiao-Hui et al. also found oridonin can induce apoptosis through Akt and MAPKs signaling pathways and inhibit the progression of OS [45, 46]. We next further confirmed the inhibition of oridonin on OS cells by cytotoxicity, plate colony formation assays, wound-healing tests and detection of the apoptosis-related indexes in the study.

Except that, the results of KEGG enrichment analysis displayed that oridonin was associated with EGFR tyrosine kinase inhibitor resistance, platinum drug resistance, T cell receptor signaling pathway and (anti-programmed cell death 1) PD-L1 expression and (programmed death-ligand 1) PD-1 checkpoint pathway in cancer and other cancer chemotherapy-related pathways in OS cells, which suggested that oridonin might be involved in the drug resistance of OS. Studies had found that the effect of oridonin on drug resistance in OS cells might related to the synergistic pro-apoptotic effect of oridonin and these chemotherapy drugs [12, 47, 48]. Liliya et al. have illustrated that oridonin could promote apoptosis, enhance the chemotherapy effect of doxorubicin in OS and reduce the clinical dosage of doxorubicin significantly. Given that the etiology of OS is complex, where multiple cell death mechanisms involved in OS’s pathological process [49]. It appears likely that the effective OS inhibitors should target multiple cell death processes simultaneously, which could also help address the problem of drug resistance [50]. In addition to apoptosis, there might exist other mechanisms for oridonin to reverse drug resistance in OS cells, which need to be further explored.

In addition, as a new type of PCD, ferroptosis had manifested as an imbalance of iron homeostasis, accumulation of ROS, mitochondrial malfunction, and so on, which plays a key role in tumor inhibition and reversal of chemotherapy, targeted therapy resistance and immunotherapy resistance [16, 23]. For example, inhibition of STAT3 could regulate GPX4, SLC7A11 and FTHI to induce ferroptosis, which can restore the sensitivity of gastric cancer cells to chemotherapy drugs [50]. Tuo et al. found that ferroptosis could overcome the resistance to EGFR-tyrosine kinase inhibitors [51]. Meanwhile, overcoming the immune checkpoint inhibitors resistance remained a great challenge in the treatment of cancers. Studies have shown that ferroptosis was involved in T cell immunity and sensitized resistant tumors to anti-PD-1 therapy [34, 35]. However, it is still unclear about whether ferroptosis was involved in the anti-OS activity of oridonin. In this study, the results showed that oridonin could reduce the expression of SLC7A11, GPX4, and FTH1, while increasing the expression of ACSL4. Furthermore, compared with the control group, oridonin could promote the production of ROS, increase Fe^2+^ level and reduce mitochondrial membrane. In addition, ferroptosis inhibitor Fer-1 could reverse the drop in ferroptosis-related indices and significantly alleviate the toxic effect on OS cells caused by oridonin. To sum up, the study was first to demonstrate that oridonin inhibits OS in a ferroptosis-depend way.

Ferroptosis and apoptosis are two types of programmed cell death, involved in ferroptosis and apoptosis were extremely essential to the growth of different tumors, which also conferred resistance to anticancer drugs [52–54]. Targeting ferroptosis and apoptosis pathways were potential therapeutics for cancer, many medicines had been reported to play anti-cancer activities via the combination routes [55]. According to the study, oridonin exhibits an anti-OS activity might via a combination of ferroptosis and apoptosis, which might be an effective approach to inhibit the development and alleviate drug resistance in OS cells.

However, there are still some limitations in this study. Firstly, drug-resistant OS cell lines have not been established in the study. Secondly, the effects of oridonin on apoptosis and ferroptosis of drug-resistant OS cell lines have not been explored. This will be the focus of our next research direction.

## Conclusion

In conclusion, the study confirmed that oridonin could inhibit the apoptosis and ferroptosis pathway of OS cells to restrict the growth and metastasis of OS which provided a novel strategy for coordinating the action of several cell death modes in the treatment of OS. Meanwhile, it provided a solid fundation for promoting the application of oridonin in clinical treatment as an antitumor drug and adjuvant chemotherapy drug.

### Supplementary Information

**Additional file 1.** WB results.

**Additional file 2:** **Table S1.** Primer sequences for q-PCR.

## Data Availability

No datasets were generated or analysed during the current study.

## Abbreviations

OS: Osteosarcoma
PCD: Programmed cell death
Fer-1: Ferrostatin-1
BCL-2: B-cell lymphoma-2
BAX: BCL2-Associated X
cl-caspase3: Cleaved Caspase-3
SLC7A11: Solute Carrier Family 7 Member 11
GPX4: Glutathione peroxidase 4
FTH1: Ferritin heavy chain 1
ROS: Reactive Oxygen Species
PD-L1: Anti-programmed cell death 1
PD-1: Programmed death-ligand 1
