# Supplementary figures and images for: Oridonin-induced ferroptosis and apoptosis: a dual approach to suppress the growth of osteosarcoma cells
Source: BMC Cancer. 2024 Feb 12;24:198. doi: 10.1186/s12885-024-11951-1 (PMC10863210; doi:10.1186/s12885-024-11951-1)

**WB results:**

**Figure 2.C**


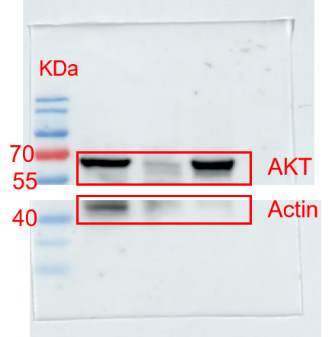


**Figure 2.D**


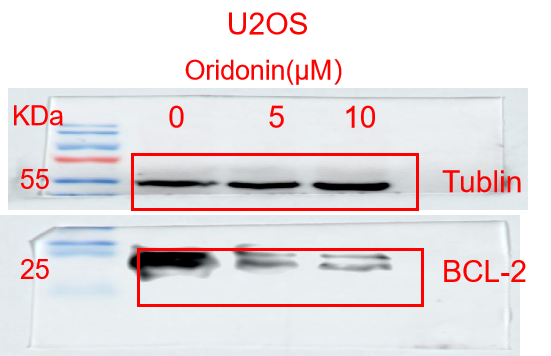

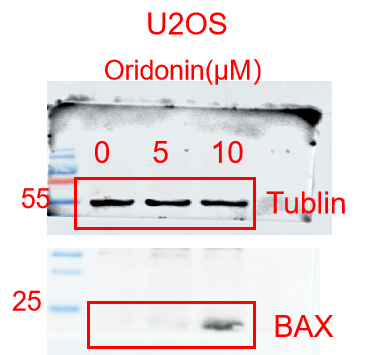

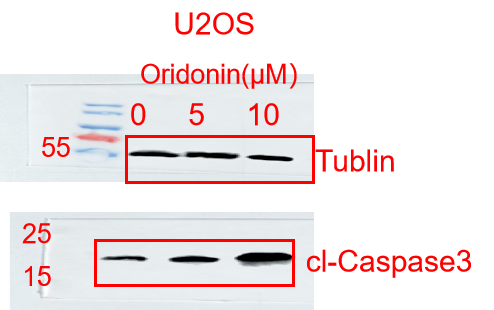


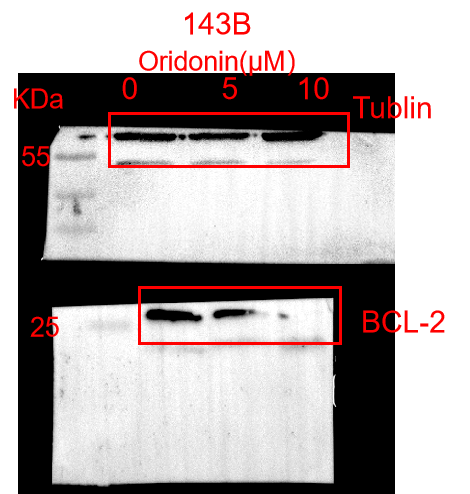

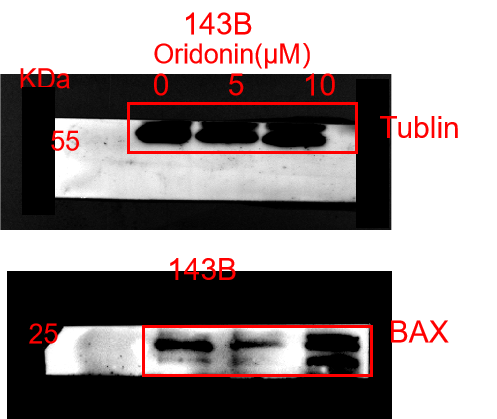

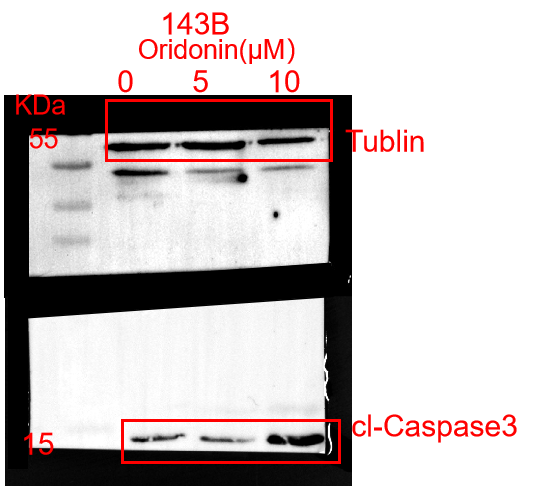


**Figure 3.A**


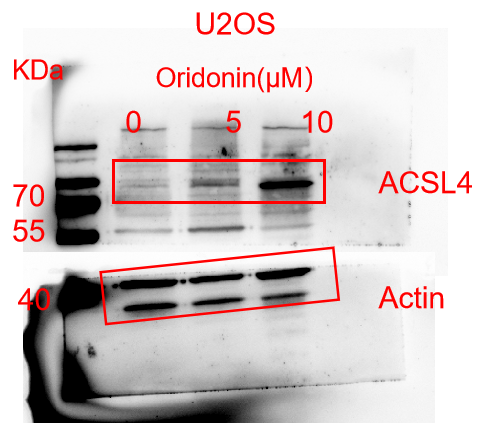

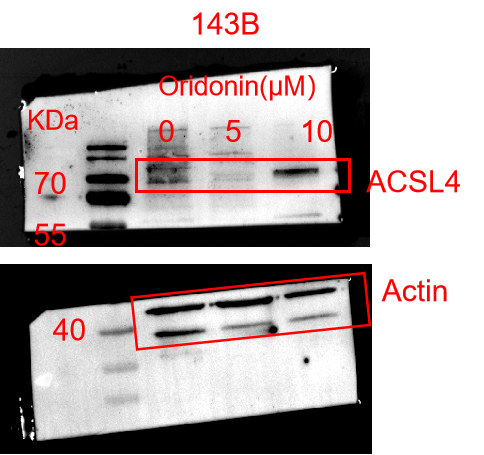

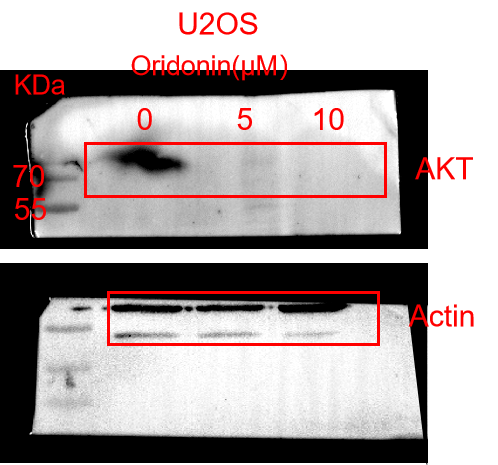


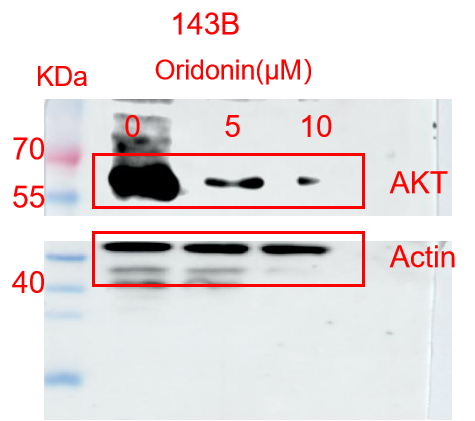

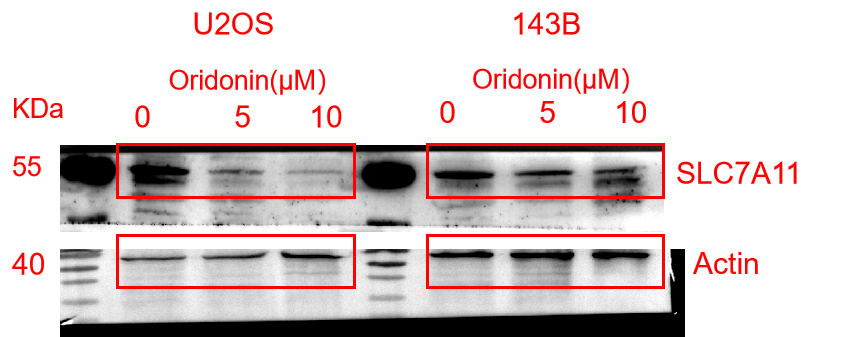


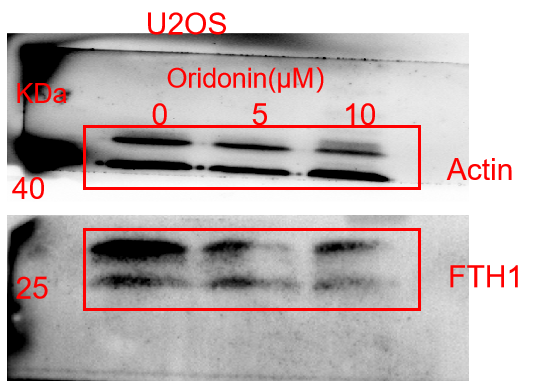

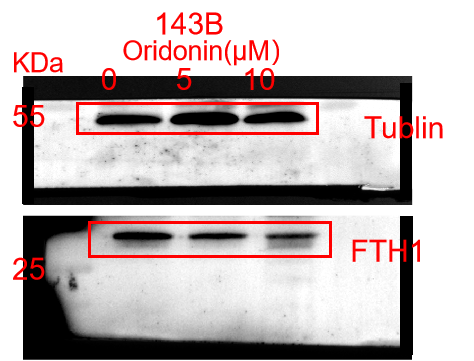


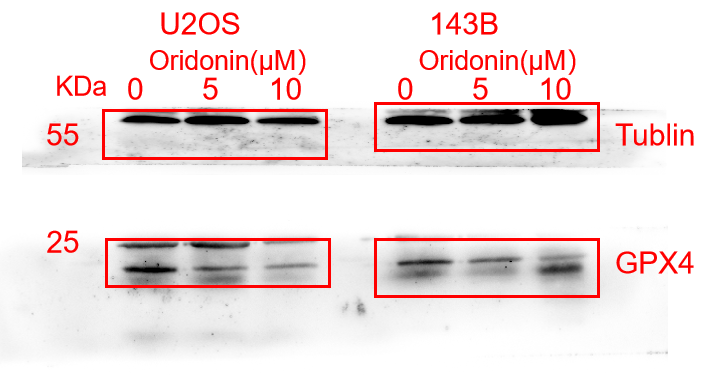


**Figure 4.A**


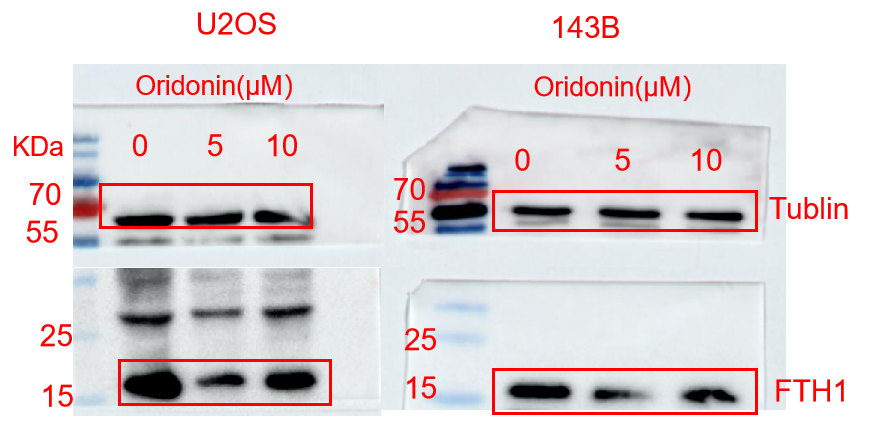

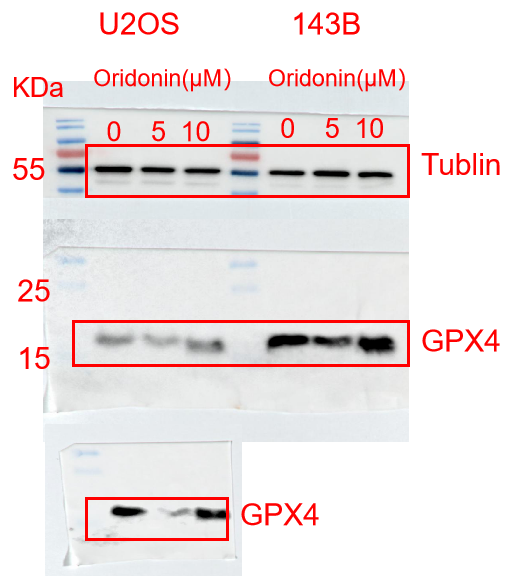

Supplement: Supplementary file 1 — Additional file 1. WB results. [file 12885_2024_11951_MOESM1_ESM.docx]
