# Supplementary material for: Oridonin-induced ferroptosis and apoptosis: a dual approach to suppress the growth of osteosarcoma cells
Source: BMC Cancer. 2024 Feb 12;24:198. doi: 10.1186/s12885-024-11951-1 (PMC10863210; doi:10.1186/s12885-024-11951-1)
Supplement: Supplementary file 2 — Additional file 2: Table S1. Primer sequences for q-PCR. [file 12885_2024_11951_MOESM2_ESM.docx]

| Genes | Forward | Reverse |
| --- | --- | --- |
| Bax | 5’-ATGTTTTCTGACGGCAACTTC-3’ | 5’-AGTCCAATGTCCAGCCCAT-3’ |
| Bcl-2 | 5’-ATGTGTGTGGAGACCGTCAA-3’ | 5’-GCCGTACAGTTCCACAAAGG-3’ |
| Cleaved  caspase3 | 5’-CTATAAGGACCTGACGGAAG-3’ | 5’-TGGAGAGGAAGATGTGAGTA |
| ACSL4 | 5’-GTTGGTCTACTTGGAGGAACG-3’ | 5’-CCTGAGGGGCTTGAAATTCAC-3’ |
| GPX4 | 5’-TGTGCATCCCGCGATGATT-3’ | 5’-CCCTGTACTTATCCAGGCAGA-3’ |
| GAPDH | 5’-AGGTCGGTGTGAACGGATTTG-3’ | 5’-GGGGTCGTTGATGGCAACA-3’ |
| SLC7A11 | 5’-TCTCCAAAGGAGGTTACCTGC-3’ | 5’-AGACTCCCCTCAGTAAAGTGAC-3’ |
| FTH1 | 5’-CCCCCATTTGTGTGACTTCAT-3’ | 5’-GCCCGAGGCTTAGCTTTCATT-3’ |
| AKT | 5’-CAACAACGCCTTCTTCCT-3’ | 5’-TCCTTCTTCAATCGCTTCAT-3’ |

**Supplementary Table**

**Table 1 Primer sequences for q-PCR**
